# Biopsychosocial and Spiritual Implications of Patients With COVID-19 Dying in Isolation

**DOI:** 10.3389/fpsyg.2020.588623

**Published:** 2020-11-17

**Authors:** Thushara Galbadage, Brent M. Peterson, David C. Wang, Jeffrey S. Wang, Richard S. Gunasekera

**Affiliations:** ^1^Department of Kinesiology and Health Science, Biola University, La Mirada, CA, United States; ^2^Rosemead School of Psychology, Biola University, La Mirada, CA, United States; ^3^Southern California Permanente Medical Group, Department of Infectious Diseases, Anaheim, CA, United States; ^4^Department of Chemistry, Physics, and Engineering, Biola University, La Mirada, CA, United States

**Keywords:** Coronavirus, death, spiritual, end-of-life, isolation, biopsychosocial-spiritual, palliative care, family

## Abstract

Critically ill patients with the Coronavirus disease 2019 (COVID-19) are dying in isolation without the comfort of their family or other social support in unprecedented numbers. Recently, healthcare teams at COVID-19 epicenters have been inundated with critically ill patients. Patients isolated for COVID-19 have had no contact with their family or loved ones and may have likely experienced death without closure. This situation highlights concerns about patients’ psychological and spiritual well-being with COVID-19 and their families, as they permanently part ways. While palliative care has advanced to adequately address these patients’ needs, the COVID-19 pandemic presents several barriers that force healthcare teams to deprioritize these essential aspects of patient care. The severe acute respiratory syndrome (SARS) outbreak in 2003 gave us a glimpse of these challenges as these patients were also isolated in hospitals. Here, we discuss the importance of the biopsychosocial spiritual model in end-of-life care and its implications on patients dying with COVID-19. Furthermore, we outline an integrative approach to address the unique and holistic needs of critically ill patients dying with COVID-19. These include intentional and increased coordination with trained palliative care staff, early and frequent goals of care including discussion of end-of-life plans, broader use of technology to improve connectedness, and shared decision making with patients’ families.

## Introduction

Sunday, May 24th, 2020, The New York Times dedicated their entire front page to remember the first 100,000 Coronavirus disease 2019 (COVID-19) related deaths recorded in the United States (United States; NYTimes, 2020). This milestone was a somber reality check, especially considering that according to the 2019 Global Health Security Index, the US has been ranked first with a national emergency preparedness index score of 83.5 (1st out of 195 countries; GHS Index, 2019). Yet, as the most prepared country, less than 3 months following the first documented COVID-19-related death in the state of Washington, the US reached a grave reality mourning for these lives lost with a case-fatality rate of 5.9% (CDC, 2020; JHU, 2020). Ultimately, this would suggest that, regardless of index positioning, no country was adequately prepared for COVID-19.

During the rapidly changing and often chaotic initial responses to the rapid influx of patients, end-of-life spiritual and psychological needs of patients and their families may have been deprioritized. Many patients with COVID-19 that died in hospitals or healthcare facilities, especially in the state of New York, died in isolation, among strangers, unable to be comforted by family and loved ones. A disturbing pattern was observed among patients who were dying haplessly in isolation in China, Italy, Spain, the United States, and now in Brazil, as the epicenter for COVID-19 has migrated westward. This situation intensified as local hospitals were pushed to their limits serving the influx of critically ill patients with COVID-19 while imposing contact and isolation precautions to prevent further disease spread. These rigorous isolation precautions directly impacted dying patients and their loved ones as both parties lost their rights to properly observe their end-of-life rites and rituals (Mayland et al., 2020).

There are three main entities involved when taking care of patients with life-threatening diseases. They include the patient, their family (including close friends), and the professional healthcare team. In terms of palliative care policy, while some discrepancy exists among the implementation of protocols in the US, hospital systems should incorporate pain and symptom management, familial support, and the communication of goals and planning of advanced end of life care (Hawley, 2017). Ultimately, the focus should be on patient quality of life, which may or may not have embedded religious or spiritual care components. Arya et al. (2020) noted that substantial patient care changes need to occur during a viral pandemic. For example, increases in the critical hospital infrastructure, including COVID-19-prepared treatment facilities, equipment, and personnel, are necessary. Shared decision making, while a core aspect of standard palliative care, maybe catastrophically impacted because of public health directives (travel and visitor restrictions), which may consequentially impact the spiritual, psychological, emotional, and social needs of the patient due to isolation away from their social circle (Arya et al., 2020).

As a matter of ethical consideration, increased social interaction, mutual respect between the patient and practitioners, and mutual transparency are essential aspects of care. Indeed, such values may already be naturally reflected in many parts of the world, in which the care system is centered more on solidarity than on the provision of care services organized with industrial criteria. However, in other contexts, firm adherence to formal protocols for end-of-life care from the point of triage throughout bereavement is emphasized. Such protocols are understood to allow for the most appropriate treatment to be imparted, thereby improving the quality of life and easing of passing (Myatra et al., 2014). In such contexts, a more holistic and integrated vision of effective end-of-life care may better serve the patient’s needs and help facilitate emotional closure to the family following the death of the patient. While caring for dying patients is not unprecedented, practices may differ based on the disease presentation and associated challenges. An example of challenging situations includes infection units or hospitals with restricted access, isolation precautions, and restrictions on after death and burial practices. These measures were implemented during highly contagious epidemics, including the severe acute respiratory syndrome (SARS) outbreak in 2003 (Shaw, 2006). As we have illustrated in this article, COVID-19 has presented some unique circumstances that have impacted the appropriate delivery of effective palliative care protocols.

## End-of-Life Care Challenges During the 2003 SARS Outbreak

When a patient is diagnosed with a life-limiting disease, whether they subscribe to a belief system or not, specific protocols are often followed to ensure the biopsychosocial and spiritual needs of patients and their families are met (Puchalski, 2007; Cook and Rocker, 2014). These protocols may often include religious rites or traditions unique to the particular belief system. In this setting, patients are typically assigned a palliative care team to work with the patient, their family, and the primary care team to prioritize a shared-decision making approach. Patient’s pain management, comfort, mental health, religious and spiritual needs, legal paperwork, and last will, burial preferences all discussed through a systematic method (Hallenbeck, 2005; Richardson, 2014). This allows the healthcare team to foster a caring attitude, uphold the patient’s autonomy, respect cultural or traditional considerations, and have open communication with the patient and their family. However, challenges to the provision of holistic patient care were reported to occur during the SARS outbreak in 2003. These included patient isolation, quarantine of contacts, restricted contact with family members, and limitation of contact with the deceased body, making it challenging to observe death rituals and funeral rites (Leong et al., 2004). In a quantitative analysis to determine the spiritual and psychosocial impacts of isolation, Leong et al. (2004) identified four emergent thematic elements in addressing the psychosocial and spiritual challenges that patients, families, and healthcare workers encountered. They were isolation, the uncertainty of the disease’s nature, sufferer caring for the suffering, and the disruption of bereavement. Patients with SARS felt isolated as they experienced an interruption of connectedness, loss of self-esteem, perceived themselves as prisoners, and a loss of health-related decisional autonomy. Patients’ families experienced isolation as they had restrictions in visiting and physically connecting with their loved ones, as all interaction was conducted through glass panels. The uncertain nature of the disease led to the development of depression, anxiety, and anger among SARS patients, as they did not know whether they needed to prepare to die or not. When death occurred suddenly and unexpectedly, families lost the opportunity to exchange final words. Families that lost their loved ones to SARS experienced a disruption of mourning as they had to adhere to state-mandated burial or cremation practices. At times they were unable to pay their last respects, felt a lack of closure, and often saw this as an undignified death (Leong et al., 2004). While there are distinct differences between the SARS 2003 and COVID-19 pandemic, similar observations of patents and families experiencing end-of-life challenges have been reported. Communication between patients with COVID-19 and their families is often exclusively virtual, and clinicians step into comfort patients close to death (Wakam et al., 2020).

## Biopsychosocial-Spiritual Model for the Care of Patients at the End-of-Life

A biopsychosocial-spiritual model is a holistic approach that acknowledges the interaction between physical, psychological, social, and spiritual aspects to patient care and patient well-being (Beng, 2004). Patients are considered as beings-in-relationship, and illness is regarded as a disruptive force in the biological relationships that can impact all other relational aspects of the patient (Sulmasy, 2002). This holistic model to patient care focuses on the intrapersonal interactions of the physical body and the mind-body connection and the patient’s extra-personal relationships with the physical environment, family, friends, and communities. The biopsychosocial-spiritual model is routinely used in the clinical setting, especially in taking care of patients dying from life-threatening illnesses (Chochinov and Cann, 2005; Sheehan, 2005; Woll et al., 2008). The Joint Commission on Accreditation of Healthcare Organizations (JCAHO) requires spiritual assessment to be part of the patientcare plan. Spirituality places an essential role at the end-of-life regardless of one’s faith, as they struggle with alienation, loss of self, forgiveness, self-exploration, search for balance, self-actualization, and consonance (Williams, 2006). While the dimension of spiritual care is often under-represented in palliative care, several clinical guidelines and protocols have allowed physicians and healthcare providers to care for patients holistically (Puchalski, 2001; Puchalski et al., 2009; Delgado-Guay, 2014). However, with the uncertainties, lack of time, and resources surrounding hospitalized patients with COVID-19, such end-of-life care models are challenging to implement. These factors may leave dying patients vulnerable to additional fear and death-related insecurities.

## Role of Faith and Science in Addressing Fears and Insecurities in Patients Dying With COVID-19

According to the Kübler-Ross model for the stages of grief and dying, end-of-life is marked by a continuum of stages that indicate the process an individual undergoes before the acceptance of his-or-her mortality (Kübler-Ross, 1969). Through the process, an individual may feel fearful and anxious. This fear has been documented in patients with life-threatening diseases such as cancer, bubonic plague, or progressive congestive heart failure (McClain-Jacobson et al., 2004). Likely, COVID-19 may also be included in this list. Each person’s belief system and his-or-her worldview include aspects of faith, religion, and scientific principles. Tensions between science and faith are more commonly expressed in western society. However, this may be less likely in eastern culture. Some experts question whether tension exists between science and faith (Barmania and Reiss, 2020), especially in eastern cultures. This was not the case in the west before the greater acceptance of Darwin’s theories on evolution became mainstream in science. They argue that a scientist may pray for a loved one who might be in a critical condition or perhaps pray for scientists and researchers to find effective treatment options for a disease (such as COVID-19) or themselves in a near-death situation. Arguably, many prolific and influential scientists have been documented throughout history who have professed deeply held spiritual or religious beliefs.

Even today, an example of someone who adheres to both science and religious values is Dr. Francis Collins, the current Director of the National Institutes of Health (NIH). He is reportedly working “all his waking hours” to find a vaccine for COVID-19 (Barmania and Reiss, 2020). In May 2020, Collins was awarded the prestigious Templeton Prize, which is bestowed on those working toward bridging the gap between faith and science. In his book *The Language of God*, Collins writes that he is a Christian who followed a path from atheism to following Jesus as a medical student when he encountered death daily (Barmania and Reiss, 2020). Holding religious beliefs may, in fact, better equip individuals managing or coping with health issues or, in the case of Dr. Collins, the tireless efforts to find effective treatments for COVID-19. As an example of this, a survey of approximately 100 patients with an end-stage disease indicated that properly functioning religious worldviews provided comfort by buffering the individual against death-related concerns (Edmondson et al., 2008). Lack of faith, reportedly, leaves the patient vulnerable to depression and the terror of death. In contrast, properly functioning faith in religion offered comfort to the individual against the concerns of death.

## Impact of Losing a Loved One to COVID-19

When patients face life-threatening illnesses such as COVID-19, family and loved ones also suffer alongside them. Insomnia, worry, concerns with treatment, a limited sense of freedom, and cultural or traditional challenges may be negatively impacted by terminal diagnoses (Golics et al., 2013). These concerns are not self-limited and affect family members’ relationships, work, education, the outlook in life, and social interactions. Another long-term implication of losing a loved one suddenly is a prolonged phase of mourning. The additive nature of loss is also significantly impacted by the uncertainty surrounding the illness, disruption of connectedness, and other factors influencing bereavement outcomes (Mayland et al., 2020). The surrounding uncertainty of the patient’s prognosis and perception of disease severity may negatively impact a family’s capacity to prepare for the loss of a loved one adequately and deliver a proper final goodbye. This uncertainty may be accentuated during pandemics, where the contagion potentially may simultaneously infect multiple family members, leaving only a couple of healthy family members to face this situation alone.

It is essential to assess the various struggles family caregivers experience appropriately and have an active support system through the grief and bereavement (Holtslander, 2008; Zaider and Kissane, 2009). Spiritually and religion play a critical role as patients and families battle end-of-life situations. Negative religious coping results in worse mental health outcomes and suboptimal quality of life in patients with life-limiting diseases (Hebert et al., 2009; Taheri-Kharameh et al., 2016). In contrast, positive religious coping has the opposite effect. The more opportunities the hospital creates for the family members to connect with their dying loved ones and offering psychosocial and spiritual support through the grieving process, they have better outcomes during their grieving process (Miyajima et al., 2014; Mayland et al., 2020).

The COVID-19 pandemic has placed a varying degree of restriction on funeral practices of patients that die from the disease. Funeral practices, including planning a meaningful funeral, memorializing lost one, mourning the collective loss, and the ability to personalize the funeral shown to have an impact on how the family received closure or went through the bereavement process (Burrell and Selman, 2020). Social distancing practices, including the inability to gather in masses, stay at home orders, and facemask requirements, have also negatively hampered families’ ability to personalize this experience (Jalali et al., 2020). The ongoing pandemic further complicates the grieving process as families have to do so in isolation, without face-to-face mourning rituals, financial uncertainties, and not being able to plan for the end-of-life care of their loved ones (Carr et al., 2020).

## Primary Care Teams Address the Needs of Dying COVID-19 Patients

While patients are dying with COVID-19 in isolation without family in hospital settings, they are not entirely alone. These patients are surrounded by well-intending physicians, nurses, and ancillary staff dedicated to COVID-19 units in the hospital. They are present for the patients in their last moments of life to make sure they are comfortable, and their death is not a painful one (Lai et al., 2020; Wakam et al., 2020). These primary care teams often take the role of hospital chaplains and palliative care teams, as limited personal protective equipment (PPE) and isolation precautions have limited these services. Primary care teams may organize and function somewhat differently in countries using insurance-based healthcare compared to thought funded by the state. In response to the COVID-19 pandemic, individual hospitals and clinical facilities have developed innovative palliative care toolkits unique to their situation and location. These may incorporate COVID-19-related apps, one-page summaries with protocols to care for dying patients, pocket cards, communication skills videos, and COVID nurse resource lines (deLima Thomas et al., 2020). These measures are indeed beneficial to the healthcare teams, patients with COVID-19, and their families as they navigate uncharted waters with the perils of COVID-19. Besides these COVD-19 specific end-of-life tools, a recent study highlighted the importance of involving palliative care teams to serve the psychological and spiritual needs of patients and families (Obata et al., 2020). As simple as it sounds, the coordinated efforts between established branches of healthcare can better address the holistic needs of COVID-19 patients. Additionally, nationwide measures in the US have been implemented to enhance care planning for dying patients, having family more involved in patient care, virtual funeral services, and remote counseling to help to grieving loved ones adapt to their loss (Carr et al., 2020; Mayland et al., 2020). The emphatic and personalized care the primary care team provides to patients in isolation has indeed made a difference in the lives of their patients.

## Implications of COVID-19 Patients Dying in Isolation

Attending to end-of-life patients’ psychological and spiritual needs is an essential component of holistic patient care (Puchalski, 2001; Sulmasy, 2002; Rego and Nunes, 2019). Besides attending to patients’ physical and medical needs with COVID-19, these aspects need to be addressed as well. In comparing the implications of isolation precautions implemented during the SARS outbreak and the COVID-19 pandemic, we can better understand the biopsychosocial spiritual needs of patients dying with COVID-19 and their families. If these needs are not adequately met, we are doing a severe disservice to dying patients who should not have to spend his-or-her final moments on earth in isolation. Not only this, but by not allowing families the right to appropriately grieve the loss of their loved one(s) may inflict consequential damage that may lead to prolonged societal dysfunction (Mayland et al., 2020).

Over the past few decades, palliative care has evolved to be instrumental in meeting the psychological and spiritual needs of end-of-life patients and their families (Puchalski et al., 2009). Psychologists and trained palliative care providers play and crucial role in providing support and comfort for patients with life-threatening illnesses and their families as they navigate these uncertain times (Rego and Nunes, 2019). However, the COVID-19 pandemic presents a unique situation in that it gives a much shorter window time to care for critically ill patients before they lose their decision-making capacity (e.g., on ventilators) or die from the disease. While health care providers have stepped up to care for patients’ needs beyond their physical illness, they are not experts in palliative care and may likely fail to sufficiently address the psychological and spiritual needs of dying patients and their families.

## Addressing the Biopsychosocial and Spiritual Needs of Patients Dying With COVID-19

**FIGURE 1:**
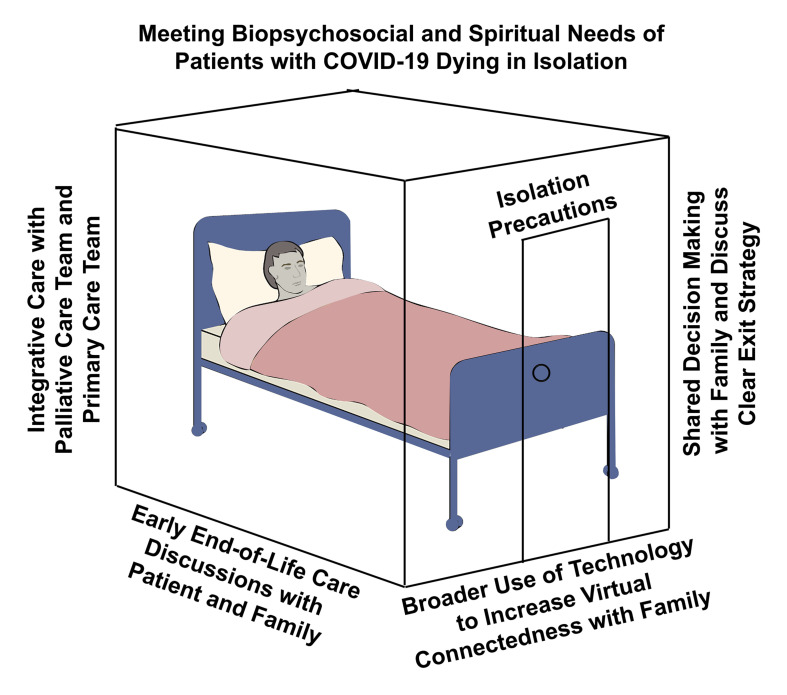
Meeting biopsychosocial and spiritual needs of patients with COVID-19 dying in isolation. Four measures to help meet the psychological and spiritual needs of patients and their families include: (1) An integrative care approach with palliative care and primary care teams working together. (2) Early end-of-life care discussions with the patient and their family. (3) The broader use of video conferencing technology within isolation precaution units to facilitated virtual connectedness with family. (4) Increased shared decision making with family and discussions about a clear post-COVID-19 exit strategy (discharge or death).

There are several measures that healthcare teams can take to effectively manage the biopsychosocial and spiritual needs of critically ill patients with COVID-19 (Figure 1). The most crucial action is to increase coordination efforts with hospital palliative care teams toward an integrative care approach during this pandemic. This approach will help effectively address most end-of-life needs by the experts. An empirical study conducted at a New York City medical center treating patients with COVID-19 showed that the involvement of palliative care teams significantly improved end-of-life care (Obata et al., 2020). Palliative care teams fostered patient autonomy helping to clarify and implement advanced directives more effectively. While isolation precautions and limited resources may be barriers, remote support from these end-of-life experts can be equally or more effective, as they can consult more patients.

The second important measure is to discuss end-of-life plans with critically ill patients and their families very early in the hospitalization process. While death is not a certainty at the time of hospitalization for COVD-19, given the erratic nature of the disease prognosis, this helps prepare patients for a probable outcome. This action will allow patients and their families to consider important spiritual aspects, including forgiveness, life after death, and provide time for closure. Third, the broader use of video conferencing technologies is an equally important practical application that can facilitate a sense of virtual connectedness between patients in isolation and their families. Virtual meetings can help remove the physical distance and visitation barriers to provide more contact time with their loved ones. Combining this with early discussions about end-of-life plans will direct families to have meaningful spiritual conversations before the death of their loved one. Hospitals caring for patients with COVID-19 can foster this by increasing the accessibility to such technology and building them into isolated COVID-19 units.

Finally, expanding shared decision-making opportunities and involving family members in patient care decisions will address their concerns and formulate a clear post-COVID-19 exit strategy. This exit strategy will cover patient outcomes, including complete recovery, debilitating conditions, longer-term care, and the death of the patient. All these action steps work synergistically toward the common goal of addressing the psychological and spiritual needs of patients dying in isolation. An integrative approach incorporating these four actions in clinical settings will facilitate holistic care for patients dying with COVID-19 and their families.

## Author Contributions

All authors listed have made a substantial, direct and intellectual contribution to the work, and approved it for publication.

## Conflict of Interest

JW was employed by company Southern California Permanente Medical Group. The remaining authors declare that the research was conducted in the absence of any commercial or financial relationships that could be construed as a potential conflict of interest.
